# Nanomaterials: A powerful tool for tumor immunotherapy

**DOI:** 10.3389/fimmu.2022.979469

**Published:** 2022-08-22

**Authors:** Ziyin Chen, Ziqi Yue, Ronghua Wang, Kaiqi Yang, Shenglong Li

**Affiliations:** ^1^ Clinical Medicine, Harbin Medical University, Harbin, China; ^2^ Department of Forensic Medicine, Harbin Medical University, Harbin, China; ^3^ Department of Outpatient, Dongying People’s Hospital, Dongying, China; ^4^ Department of Bone and Soft Tissue Tumor Surgery, Cancer Hospital of Dalian University of Technology, Cancer Hospital of China Medical University, Liaoning Cancer Hospital and Institute, Shenyang, China

**Keywords:** nanomaterials, anti-tumor, immunotherapy, tumor microenvironment, immunosuppression

## Abstract

Cancer represents the leading global driver of death and is recognized as a critical obstacle to increasing life expectancy. In recent years, with the development of precision medicine, significant progress has been made in cancer treatment. Among them, various therapies developed with the help of the immune system have succeeded in clinical treatment, recognizing and killing cancer cells by stimulating or enhancing the body’s intrinsic immune system. However, low response rates and serious adverse effects, among others, have limited the use of immunotherapy. It also poses problems such as drug resistance and hyper-progression. Fortunately, thanks to the rapid development of nanotechnology, engineered multifunctional nanomaterials and biomaterials have brought breakthroughs in cancer immunotherapy. Unlike conventional cancer immunotherapy, nanomaterials can be rationally designed to trigger specific tumor-killing effects. Simultaneously, improved infiltration of immune cells into metastatic lesions enhances the efficiency of antigen submission and induces a sustained immune reaction. Such a strategy directly reverses the immunological condition of the primary tumor, arrests metastasis and inhibits tumor recurrence through postoperative immunotherapy. This paper discusses several types of nanoscale biomaterials for cancer immunotherapy, and they activate the immune system through material-specific advantages to provide novel therapeutic strategies. In summary, this article will review the latest advances in tumor immunotherapy based on self-assembled, mesoporous, cell membrane modified, metallic, and hydrogel nanomaterials to explore diverse tumor therapies.

## Introduction

As a significant malignant disease endangering human health, there were 10 million deaths in 2020 alone (1). Although cancer treatment strategies have evolved from the initial single surgical treatment to a combination of radiotherapy and chemotherapy, the high mortality rate and poor prognosis associated with cancer have been a social problem worldwide. As research progresses, the immune system’s role in oncology is emerging. Dysregulation of the immune system results in multiple diseases, including cancer. Early studies suggested that the immune system primarily prevents tumors by eliminating or suppressing viral infections, eliminating pathogens, clearing inflammation, and identifying and removing tumor cells based on tumor-specific antigens. As research progresses, the immune system has also shown its relevance in cancer development, progression and resistance to drugs. Tumor cells interact with the immune system to provide a tumor microenvironment (TME) that is more suitable for cancer cells to survive (2, 3). Cancer cells achieve immunosuppression through various mechanisms, including secretion of cytokines and upregulation of immune checkpoint receptor ligands. In this process, immunosuppression is also achieved by training immune cells, which leaves the TME in an immunosuppressed state (4). Immune cells interact with tumor cells in the TME to regulate and thus promote cancer growth and immune evasion (5, 6). Based on the above background, cancer immunotherapies are represented by immune checkpoint blockade (ICB), chimeric antigen receptor-T cell (CAR-T), and neoantigen vaccines have emerged (7–10). They have transformed conventional cancer treatment and demonstrated the potential of using the body’s defense system to fight cancer (11).

**Scheme 1:**
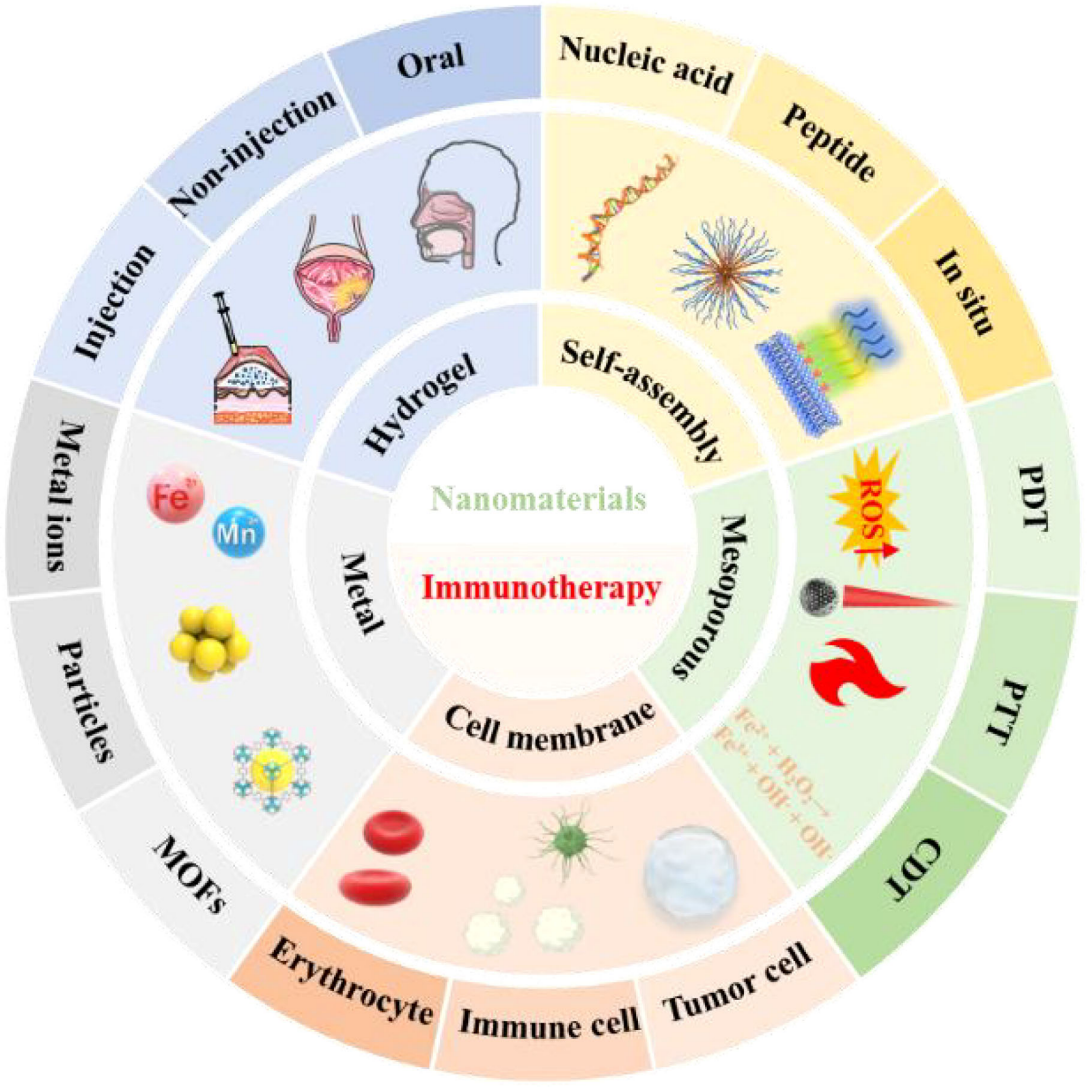
Schematic diagram of nanomaterials based on different structures, functions and delivery modes for antitumor immunotherapy applications. Immunotherapy can be customized according to tumor characteristics as a precision therapy strategy. However, for some patients who can still not benefit from the results, the nanomaterial-based immunotherapy strategy has delivered a satisfactory answer. Nucleic acids and peptides with self-assembly functions can affect and regulate the body's immune function through exogenous introduction and interference. Mesoporous materials have strong loading capacity and modifiability. They can achieve the purpose of tumor immunotherapy through the synergistic effect of multiple treatment regimens (PTT, PDT, CDT, etc.) in the case of loading or not loading drugs (or photosensitizer, etc.). To prevent tumor cells from rejecting the intruder, nanomaterials can be disguised (tumor cell membrane modification, erythrocyte membrane modification, etc.) to achieve the purpose of entering the tumor. A suitable nanostructure modification can release the drug by receiving a specific trigger at a targeted site (immune organs, tumor tissues, etc.). The diversity of endogenous (pH, ROS, GSH, etc.) and exogenous (light, ultrasonic, etc.) trigger devices makes nanomaterials controllable in time and space. At the same time, according to the different tumor sites of patients, a personalized drug administration plan (injection, non-injection, oral) can improve patient compliance and achieve the effect of longterm induction of immunity.

Tumor immunotherapy has emerged as a highly sought-after innovative oncology treatment option and has become the fourth cancer treatment alongside surgery, chemotherapy and radiotherapy (12, 13). Although these therapies have shown promising clinical performance in some populations, the number of beneficiary groups is limited due to several factors. Most solid tumors are not as well treated. These effects include, but are not limited to, differential response rates across tumors, heterogeneity in antigen expression, antigen-negative hosts, and an immunosuppressed tumor environment (14, 15). Improving the success rate of immunotherapy and reducing the side effects of treatment-induced autoimmune diseases has become a significant challenge. Nanomaterial-based delivery strategies have delivered a satisfactory answer to the critical issues in cancer immunotherapy. Nanomaterials can achieve targeted drug delivery to tumor sites or immune organs due to their advantages of large modifiable functional groups and drug-carrying capacity (16, 17). By responding to endogenous or exogenous stimuli out of their flexible versatility to achieve special functions for drug integration, efficient penetration of biological barriers, precise delivery of immunomodulators, and controlled release, thereby facilitating tumor immunotherapy (18, 19). Herein, we review the latest advances in self-assembly strategies, mesoporous delivery systems, biofilm delivery strategies, metallic materials, and hydrogel materials for tumor immunotherapy. In addition, we provide an outlook on this emerging field at the intersection of cancer immunotherapy and nanomaterials, as shown in **Scheme 1**.

## Self-assembled multifunctional nanomaterials

Self-assembly is the spontaneous formation of ordered structures of essential substances based on non-covalent bonding interactions rather than the simple superposition of substances (20). The unique cross-structural properties of the self-assembly process have led to its widespread use for drug delivery (21). For example, the formation of nanoparticles in the form of small molecule anti-tumor drugs by self-assembly solves the problem of the short retention time of drugs at tumor sites. Meanwhile, nanosystems based on self-assembled structures can achieve specific release of anti-tumor drugs (22). Meanwhile, small molecules arranged in self-assembly improved the low response rate and immune-related adverse reactions in cancer immunotherapy.

### Nucleic acid-based self-assembly

Nucleic acids are essential components of life and are necessary for storing and transmitting genetic information. As research progresses, the concept of functional nucleic acids (FNAs), which contain nucleic acids and nucleic acid mimetic molecules, has emerged. FNAs, including DNAzymes, nucleic acid aptamers and DNA origami, have independent structural functions and perform specific biologically non-genetic functions (23–25). Combining these substances with nanomaterials offers a new direction for anti-tumor immunotherapy, thanks to the advantages of containing easily modifiable functional groups, flexible structures, and efficient catalytic capabilities (26).

**Figure 1:**
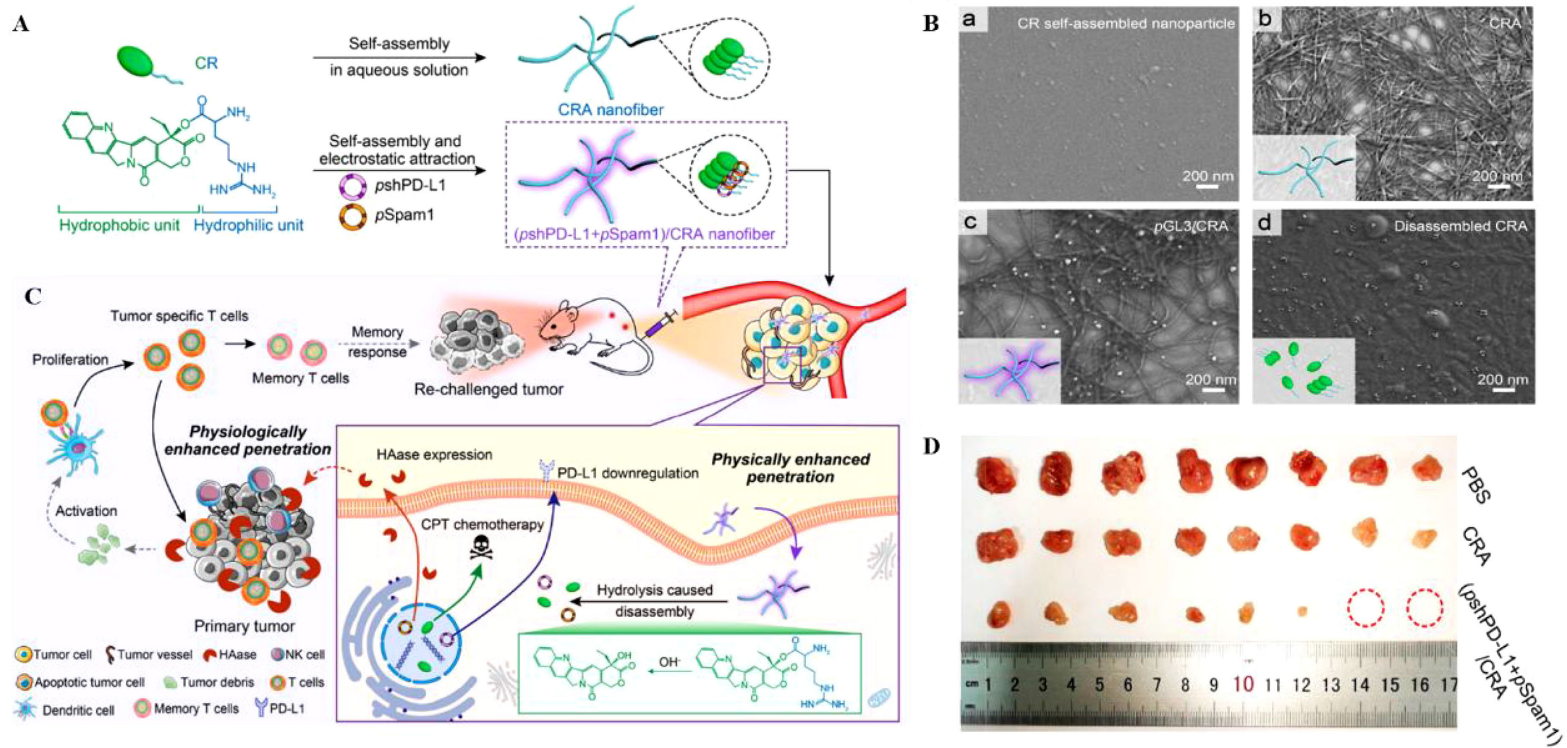
**(A, B)**. Schematic diagram of (pshPD-L1+pSpam1)/CRA self-assembly (Scale Bar = 200nm). **(C)** Schematic diagram of (pshPD-L1+pSpam1)/CRA for tumor immunotherapy. **(D)** Tumor size of 4T1 tumor-bearing mice treated with PBS, CRA and (pshPD-L1+pSpam1)/CRA, respectively (31). Reprinted with permission from Ref. (31). Copyright© 2021, copyright Guo et al.

Hyaluronan is a polysaccharide highly expressed in tumors and serves as a vital component of the ECM, supporting tumor growth in a gel state (27, 28). Hyaluronidase (HAase) regulates hyaluronan by enzymatic digestion (29, 30). Disruption of the physiological matrix barrier of the TME implies enhanced penetration of nano drugs into the tumor and increased infiltration of immune cells. Chen et al. (31) proposed a prodrug delivery strategy for specific target gene delivery to tumor sites, resulting in the construction of (pshPD-L1+pSpam1)/CRA. CR(CPT-Arginine) acts as a prodrug molecule, coupled by CPT (Camptothecin) and modified L-Arg, and forms CRA (CR self-assemble) nanofibers by self-assemble. Arginine-modified camptothecin (CPT) prodrugs self-assemble into nanofibers for enhanced tumor penetration through hydrophobic interactions, and the structure can be delivered as a gene carrier **(** **Figures 1A, B** **)**. The pshPD-L1 expresses small hairpin RNA of programmed death-ligand 1 (PD-L1) to silence PD-L1 for *in situ* immune checkpoint blockade, and pSpaml expresses HAase to degrade HA. (pshPD-L1+pSpam1)/CRA inhibited CPT-induced PD-L1 upregulation, and pSpaml reconstituted ECM to increase CD8^+^T-cell infiltration, which effectively led to the formation of memory T-cell and the maturation of DC with chemotherapy-coordinated immunotherapy **(** **Figure 1C** **)**. Ultimately it achieved better tumor suppression at the 4T1 breast cancer cell level and *in vivo* models with reduced systemic side effects. To demonstrate that this nanoplatform could be an ideal tumor immunotherapy strategy. The authors also validated the immune memory capacity after (pshPD-L1+pSpam1)/CRA treatment. 4T1 cells were injected subcutaneously into the contralateral side after immunotherapy in hormonal mice. Encouragingly, the growth of heterolateral tumor changes was significantly inhibited, with 25% of the tumors remaining ungrown on day 12 after tumor inoculation **(** **Figure 1D** **)**. This treatment strategy enhanced the anti-tumor ability by enhancing memory T cells. This nucleic acid delivery system organically combines chemotherapy with immunotherapy, overcoming the disadvantages of poor penetration, premature release and low bioavailability of chemotherapeutic drugs at tumor sites.

Synthetic CpG-ODN can mimic the immunostimulatory activity of natural CpG-DNA (32, 33). After recognition by toll-like receptor 9 (TLR9), recognition in the endosomes of antigen-presenting cells (APCs) could strengthen the activity of immune cells and promote cytokine secretion (34). Still, cellular internalization efficiency is low due to the negative charge factor of CpG-ODN and DNA endonuclease (DNase) degradation, making it difficult to achieve the desired therapeutic level (35, 36). Ding et al. (37) used an enzyme-free method to rapidly assemble controllably sized, low-cost self-assembled dendritic DNA nanomolecules. The DNA dendrimer molecules were assembled with a cyclic CpG-ODN with closed open ends on the surface, enhancing the stability of CpG while providing more efficient cellular uptake than short DNA single strands. DNA dendritic polymer molecules were modified using the cellular membrane penetrating peptide transactivator of transcription (TAT) to improve the membranous permeability of nanomaterials. The modification of TAT was used to improve the cell membrane permeability of the nanocarriers to ensure efficient accumulation of nanomaterials in the cells. End product G2-Loop-TAT showed an average diameter of 46.60 ± 3.6 nm in dynamic light scattering experiments. The cellular uptake capacity was substantially increased due to the dendritic structure of the DNA and the modification of the TAT peptide. When G2-Loop-TAT was co-incubated with RAW264.7 (a murine-derived macrophage cell line) at a density of 5 nM for 24 h, there was no cytotoxicity and even a slight stimulation of cell growth. An adequate secretion of inflammatory cytokines, e.g., TNF-α and IL-6, were detected in the medium supernatant. This is because TLR9 recognizes CpG-ODN and activates the following pathways to initiate immune effects. This approach makes DNA more resistant to endonuclease degradation. It provides precise control of immunostimulatory molecules due to high CpG loading and modification of TAT to achieve an elastic immune response. This strategy can improve the targeting ability and immunogenicity by modifying the targeting motifs and loading antigens, ultimately providing a promising alternative for immunotherapy.

### Peptide-based self-assembly

Peptides are the fundamental components of cells and tissues and play a crucial role in biological activities by forming proteins of living matter in certain combinations (38). When a peptide exerts its natural effect, it usually first binds to a homologous receptor on the target cell membrane and is subsequently dragged into the cell membrane by the receptor. This process consists of two regions: 1. the extracellular structural domain, where the peptide is located; 2. the intracellular structural domain, where the peptide functions after penetrating the cell membrane (39). Peptides usually contain 2-50 amino acid residues and can be divided into two main groups: oligopeptides containing a relatively small number of amino acids (i.e., 2-20) and peptides with long amino acid sequences; thus, their function depends on three key parameters: the properties of the amino acids, the sequence of amino acids and the shape of the peptide. Due to their excellent biocompatibility, biodegradability and ease of modification, the structures formed by different peptide assemblies can achieve low toxicity and stable drug release advantages (40). By modifying and modifying the peptides, the peptides can form various structures, including nanofibers and nanosphere vesicles. When in the solution state, the non-covalent binding is weak as the driving force for the formation of self-assembled structures of polypeptides. These interactions lead to the possibility of regulating self-assembly by adjusting factors such as temperature, pH and concentration. Although small-molecule peptides self-assemble in various forms, their self-assembly processes have the following commonalities: (1) assembly module peptides form nanostructures only at specific sites, such as target cells, organs or tissues; (2) response modules trigger the self-assembly process under specific environmental conditions rather than arbitrary conditions, such as high intracellular concentrations of reactive oxygen species (ROS) or stimulatory reaction conditions such as TME-specific enzymes; (3) at target sites the formation of nanostructures can be observed and lead to changes in cell structure or function (41, 42).

Molecular targeted therapy (MTT) is a cancer treatment that targets specific molecules on cancer cells rather than normal cells for appropriate drug intervention. It would be a significant breakthrough if it could selectively inhibit cancer cells while “heating “ the TME. EphA2, a critical member of the tyrosine kinase ephrin receptor, had a major role in regulating carcinogenesis and tumor progression. EphA2can be targeted to treat cancer by, for instance, reducing expression, promoting degradation, and blocking endogenous activation (43). Ding et al. (44) designed and synthesized a self-assembled peptide known as DBT-2FFGYSA for cancer therapy. FFGYSA was used to promote self-assembly and improve targeting ability. And double aromatic phenylalanines (FF) were used to enhance self-assembly performance. YSA was able to target tumor overexpressed EphA2 protein receptors, causing EphA2 receptors to form large clusters. The formation of such aggregates promoted cross-phosphorylation between EphA2 receptors, which in turn transduced anti-tumor signaling pathways while avoiding toxicity to normal cells. The environmentally sensitive fluorescent molecule DBT binds to the EphA2 protein receptor and generates fluorescence, a phenomenon that is absent in water. The injection of DBT-2FFGYSA into 4T1 and CT26 hormonal mouse models revealed a marked improvement in the distribution of CD4^+^T and CD8^+^T cells in tumor tissues, which could cause immunogenic cell death (ICD) of tumor cells and release tumor antigens and damage-associated molecular patterns (DAMPs). The released DAMPs assist immune cells in presenting tumor antigens to T cells and enhance T cell activation, proliferation and infiltration (45). The percentage of PD-1^+^CD8^+^T cells was dramatically reduced after DBT-2FFGYSA treatment, successfully transforming cold tumors lacking T cell infiltration into immunogenic hot tumors. A hot tumor is considered to be a tumor with a high infiltration of immune cells, in contrast to a cold tumor, which is a tumor with little infiltration and difficult penetration of immune cells. Moreover, this tumor treatment strategy was specific, with negligible harm to major organs and normal tissues, showing a promising *in vivo* safety profile. As sequencing technology develops, more and more tumor and immune cell-specific molecules are being identified as targets for tumor immunotherapy. Strategies that selectively inhibit cancer cells by targeting specific molecules and enhance immune response without harming normal tissues should receive wide attention.

**Figure 2:**
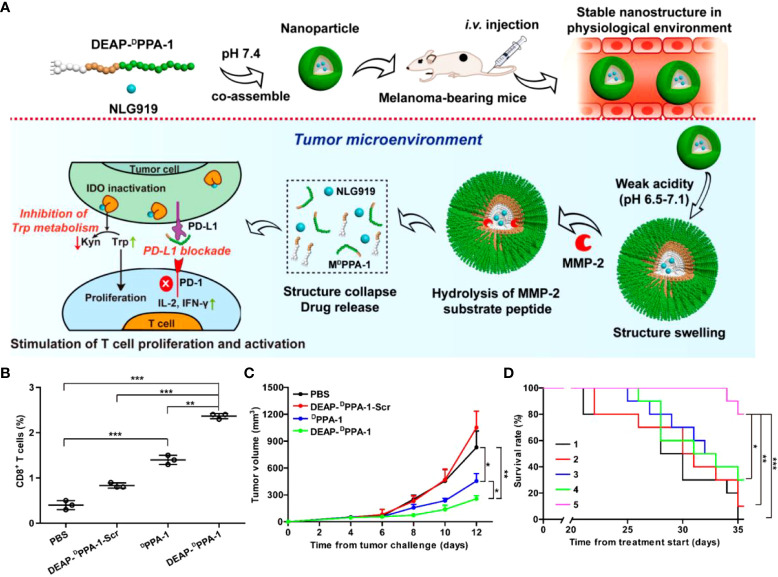
**(A)**. Schematic representation of NLG919@DEAP-^D^PPA-1 assembly *in vitro* and tumor immunotherapy *in vivo*. **(B, C)** Proportion of CD8^+^T cells in tumor tissues and tumor volume in B16-F10 tumor-bearing mice after treatment with PBS, DEAP-^D^PPA-1-Scr, ^D^PPA-1 and NLG919@DEAP-^D^PPA-1, respectively. **(D)** Survival experiment of B16-F10 tumor-bearing mice after treatment with different groups. Treatment groups: 1, DEAP-^D^PPA-1-Scr; 2, NLG919; 3, NLG919@DEAP-^D^PPA-1-Scr; 4, DEAP-^D^PPA-1; 5, NLG919@DEAP-^D^PPA-1 (*p < 0.05, **p < 0.01, ***p < 0.001) (47). Reprinted with permission from Ref. (47). Copyright© 2018, copyright Cheng et al.

PD-L1 is overexpressed in many solid tumors and inhibits kinase signaling pathways by interacting with PD-1, ultimately suppressing the proliferation and activity of T cells (46). Also, the enzyme IDO is highly expressed in tumors, leading to the degradation of L-tryptophan to L-kynurenine. Its high expression leads to tryptophan unavailability and L-kynurenine accumulation in the TME. It is also considered an effective mechanism for suppressing the effects of T cell proliferation and activity. Nie et al. (47) designed a dual-responsive multifunctional peptide assembly nanomaterial to achieve simultaneous intervention in PD-1/PD-L1 and IDO through precisely controlled drug release. This approach modified the PD-L1 antagonistic ^D^PPA-1 peptide with DEAP molecule and MMP-2 to obtain DEAP-^D^PPA-1. Then DEAP-^D^PPA-1 was co-assembled with the highly selective IDO inhibitor NLG919 to form micellar nanoparticles NLG919@DEAP-^D^PPA-1 under physiological conditions. The weak acidic pH (~6.5-7.1) unique to the TME triggers a decrease in the hydrophobicity of DEAP, which loosens the nanoparticle structure. Eventually, the nanoparticles wholly collapsed due to the hydrolysis of the substrate peptide in the presence of MMP-2 **(** **Figure 2A** **)**. NLG919 release and its co-immunotherapeutic effect were evaluated in a B16-F10 melanoma mouse model. The results showed that NLG919@DEAP-^D^PPA-1 ensured the proliferation and activation of T cells in tumor tissues **(** **Figure 2B** **)**, delayed melanoma growth **(** **Figure 2C** **)**, and significantly prolonged survival time in rheumatized mice **(** **Figure 2D** **)**. This combination immunotherapy strategy targeting the sequential response of the TME allows for precise and controlled drug release. It also enhanced the antitumor immune response by improving the stability of the drug with limited adverse effects. Regimens that target such immune targets for co-inhibition to raise immune checkpoints are often limited by factors such as poor water solubility. If adequate treatment is achieved, it comes at the cost of extremely high doses, which leads to serious adverse effects, including hypokalemia and abdominal pain. Therefore, using self-assembly strategies to improve their bioavailability and reduce their toxicity of administration addresses these bottlenecks.

### *In situ* self-assembly

Peptides composed of various amino acids have intrinsic biocompatibility, bioactivity and biodegradability, and they can construct ordered nanostructures by *in situ* assemblies in cells. Nanomaterials assembled with *in situ* self-assembly strategy as a starting point are widely used in drug delivery and cancer therapy (48–50). They can selectively respond to physiological changes or TME by targeting the tumor extracellular matrix, tumor neovascularization, cell membranes with specific receptors, and other structures (51, 52). M1-TAMs are thought to suppress tumor growth, in contrast to M2-TAMs. The latter’s functions promote tumor cell invasion, growth and metastasis. Therefore, modulating the tumor immunosuppressive microenvironment (TIME) by remodeling TAM subpopulations can help achieve immune normalization (53–55). However, achieving the goal of complete tumor eradication through cancer immunotherapy strategies with only single TAM polarization is difficult. Photoimmunotherapy (PTI) is a regimen for synergistically increasing anti-tumor immunity through photo-thermal or photo-chemical conversion (56). However, when tumor cells are treated with PTI, the self-protective mechanism of autophagy is initiated to evade immune surveillance (57).

Wu et al. (58) constructed a photothermally mediated *in situ* self-assembled nanogel (Mil) DMN using amphiphilic substances and hydrophilic hyaluronic acid (HA) as a backbone. Under normal conditions, ICD inducer photosensitizer (IR780) and autophagy inhibitor chloroquine (CQ) was co-encapsulated in the DMN. Self-assembly of C/I-Mil nano micelles formed after skin insertion and was accompanied by dissolution of DMNs. The substrate substance HA of DMNs forms C/I-Mil also through electrostatic interaction, thus enhancing tumor-targeting ability through the specific interaction of HA with CD44 receptors. C/I-Mil size regulated by photothermal could penetrate deeply into the tumor tissue and be heavily internalized by the CD44 receptor. It accurately eradicated tumors in concert with autophagy inhibitors and promoted the release of DAMPs. In addition, CQ could remodel the M1 phenotype of TAM by activating NF-κB. Using tumor-bearing mice as experimental subjects, local PTI was found to effectively eliminate primary and distant tumors with the cooperative effect of autophagy inhibition and obtained the most extended survival period by remodeling the TIME. A dual attack regimen that combines re-education of macrophages with the combat of the defense system opens another door for tumor immunotherapy.

## Mesoporous nanomaterials

Due to the lack of tumor specificity in traditional chemotherapeutic strategies, increasing the therapeutic dose often results in severe toxicity to other organ systems (59, 60). Continuous refinements of nanotechnology, including mesoporous materials, are also receiving increasing attention for biomedical applications (61). It improves bioavailability, identifies tumor cells and stays long at the tumor site—achieving enhanced therapeutic efficacy with little harm to healthy cells. Mesoporous materials can also degrade spontaneously in human tissues. Widely used and play an essential role in anti-tumor therapy (62). This section will review the application of mesoporous materials in tumor immunotherapy based on photodynamic therapy (PDT), photothermal therapy (PTT), and chemodynamic therapy (CDT).

### PDT-related mesoporous nanomaterials

As a non-invasive tumor ablation method, PDT induces tumor cell death by activating photosensitizers. With specific wavelengths of laser light to generate cytotoxic ROS (63, 64). In addition to killing cells and disrupting tumor vasculature, PDT promotes a strong anti-tumor immune response by inducing ICD. Due to its excellent therapeutic efficacy, PDT has been approved for clinical treatment (65, 66).

PDT has good therapeutic efficacy for localized tumors accessible to light sources and remains challenging in treating disseminated, metastatic cancers. Moon et al. (67) proposed to load various neoantigen peptides, CpG, and the photosensitizer chlorine e6 (Ce6) onto mesoporous silica nanoparticles (bMSN) to obtain bMSN(CpG/Ce6)-neoantigen. The nanoplatform has an average dimension of about 80 nm, with a large pore size of 5-10 nm, and is biodegradable. Notably, the bMSN (CpG/Ce6)-neoantigen can be used for diagnostic positron emission tomography (PET) imaging by loading the radioisotope ^64^Cu. It enables the monitoring of information such as the size and location of the tumor during treatment and the optimal treatment time window. In the experiment, PET images showed efficient accumulation of tumors after intravenous injection. And the tumor sites treated with this therapy effectively recruited dendritic cells (DCs) after laser irradiation. They triggered neoantigen-specific, tumor-infiltrating CTL. the anti-tumor effects of PDT immunotherapy on locally treated tumors and distal untreated ones were well demonstrated in a bilateral tumor mouse model. They suggested that bMSN could be a promising platform as a suitable PDT vector and could be combined with imaging on personalized immunotherapy surveillance to treat advanced cancers.

Light irradiation as an excitation trigger for exogenous spines has the advantages of easy and precise control, deep penetration into tissues, and little photodamage, which can prevent the adverse consequences caused by premature leakage and degradation of drugs in the natural environment (68, 69). Peng et al. (70) achieved the photo-responsiveness of mesoporous silica by introducing diselenide bonds into the framework that can be cleaved by ROS. It was loaded with photosensitizers methylene blue (MB) and Doxorubicin (DOX) and was modified with polyethylene glycol (PEG) on the surface. The results show that the ROS generated by MB under red light irradiation cleaved the diselenide-bonded silica backbone, leading to the release of MB based on matrix degradation and further generation of ROS. At the same time, this cascade reaction promotes DOX release and kills tumor cells synergistically with PDT, enhancing the effect of ICD *in vitro*. Notably, this process also induced a more robust immune response by activating NK cells. Notably, the primary tumors in mice regressed after receiving SeMSN-PEG@M&D system injections. Under red light irradiation, this chemotherapy combined with PDT also resulted in the suppression of distant tumors. It was also demonstrated that this nanoplatform combined with PD-1 checkpoint blockade therapy could achieve more significant tumor suppression. This kind of targeted drug release through exogenous stimulation, targeting the tumor site, can maximize the therapeutic effect, reduce the side effects, and effectively prevent tumor cells from resurfacing.

### PTT-related mesoporous nanomaterials

PTT uses photothermal conversion agents (PTAs) to obtain energy from near-infrared (NIR) light to trigger tumor cell death by increasing the temperature of the tumor surroundings (71, 72). However, PTT has the defects of not being able to completely eradicate and prevent the metastasis and recurrence of tumors (73). Not to be overlooked is that the drive for immunotherapy is hampered by the low immunogenicity of cancer and the existence of TIME. Therefore, protocols combining PTT with immunotherapy to improve the benefits of both have received increasing attention.

The maturation of DCs drives their function in initiating immune responses. TLR agonists induce DCs maturation and restore DCs function by activating DCs to express TLRs. DCs-rich lymph nodes are recharge stations for the body’s immunity (74, 75). Still, due to the lack of targeting, most TLR agonists are systemically dispersed after local injection, failing to form a systemic cascade of pro-inflammatory responses and causing immune-related toxic reactions. Polydopamine (PDA), a polymeric form of dopamine, is an essential neurotransmitter in signaling. Cai et al. (76) loaded TLR7 agonist R837 onto mesoporous polydopamine nanoparticles (MPDA NPs), whose photothermal conversion efficiency (η) was close to 40%. Outcomes indicated that the subcutaneous injection of PVP-MPDA@R837 NPs would enter the nearby lymph nodes, maximizing the exposure of R837 to lymph and enhancing its retention capacity, ultimately activating DCs effectively. Such effect was further strengthened by the simultaneous release of endogenous antigen with the photothermal product of MPDA, driving apoptosis of tumor cells. In the B16 melanoma model, tumor growth was effectively inhibited by thermal ablation of tumor apoptosis and cytotoxic T lymphocyte production. Overall, this nanoplatform provides a strategy for PTT combined immunotherapy targeting lymph nodes, which has great potential in tumor immunotherapy.

**Figure 3:**
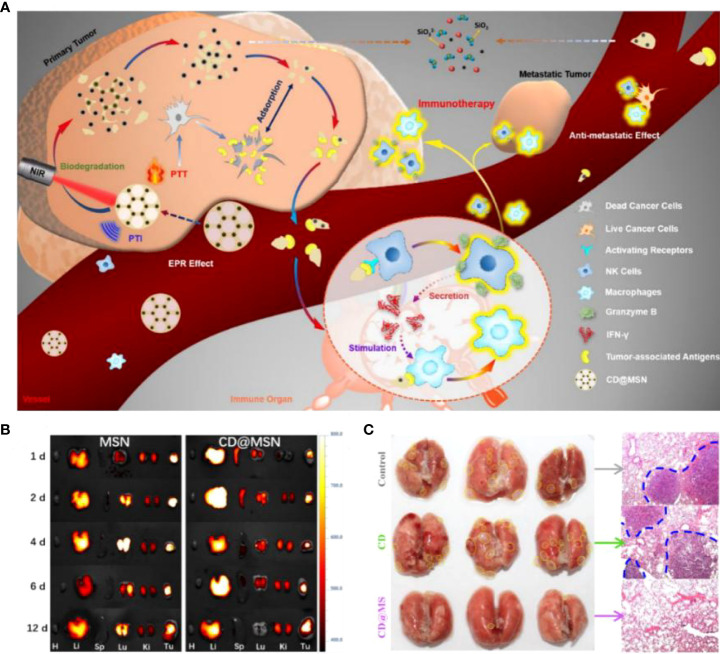
**(A)**. Schematic diagram of biodegradable CD@MSN combined with PTI and PTT for tumor immunotherapy. **(B)** Fluorescence images of major organs (heart, liver, spleen, lung and kidney) and tumors at different time points after intravenous injection of Cy5-MSN and Cy5-CD@MSN, respectively. **(C)** Photographs of the lungs of each group after 14 days of administration of different treatment regimens to the tumor-bearing mice (yellow dashed lines indicate the foci of metastatic tumors) and corresponding H&E-stained sections (blue dashed lines indicate the borders of metastatic tumors) (77). Reprinted with permission from Ref. (77). Copyright© 2019, copyright Qian et al.

Mesoporous silica nanoparticles (MSNs) have great potential for development because of their high surface area ratio, excellent drug loading capacity, and good hydrophilicity. However, the biodegradability of MSNs connected solely by the Si-O-Si framework still needs improvement. Huang et al. (77) obtained biodegradable CD@MSN by introducing carbon nanodots (CD) into the MSN framework. This strategy solved the rapid renal excretion of high-performance photothermal material CD without enrichment in tumors and advanced the application of PTT. In the experiment, biodegradable nanofragments were found to obtain tumor-associated antigens (TAAs) *in situ* from tumor cells after photothermal treatment. The nanofragments carried antigens that induced the proliferation and activation of NK cells and macrophages while increasing cytokine levels to achieve synergistic inhibition of tumor metastasis **(** **Figure 3A** **)**. Ultimately, tumor metastasis is inhibited by selective access to immune organs. The distribution of nanoparticles in the body is crucial for their metabolism and therapeutic efficacy. CD@MSN not only accumulates in large amounts at tumor sites but can also be distributed explicitly to the immune organs, spleen, liver, and kidneys. Conversely, the large amount of MSN in the lungs may lead to adverse consequences such as mechanical obstruction of capillaries **(** **Figure 3B** **)**. Notably, NIR irradiation of mice treated with CD@MSN at week 2 revealed significant inhibition of lung metastasis **(** **Figure 3C** **)**. This study presented an unconventional synthetic method for mesoporous silica and provided creative insights into the anti-cancer immunity associated with biodegradable nanoparticles.

### CDT-related mesoporous nanomaterials

Shi et al. (78) defined the treatment using Fenton or Fenton-like reactions to generate ·OH in the tumor region as CDT. Nanomaterials’ rapid development has promoted CDT use in biomedical applications as a new strategy for widely used cancer therapy with its unique advantages (79, 80). The CDT process is independent of external stimuli. It can also modulate the TME in hypoxia and immunosuppression (81, 82). In addition to the classical CDT strategy induced by iron, CDT strategies mediated by other metal elements through Fenton-like reactions have been proposed. In addition, CDT has been combined with other therapies such as chemotherapy, radiotherapy, and immunotherapy to achieve enhanced anti-cancer effects.

Tumors dramatically diminish the effect of monotherapy by domesticating TME. Lin et al. (83) used PEG to modify hollow mesoporous Cu_2_MoS_4_ (CMS) with loading glucose oxidase (GOx). The end product obtained, PEGylated CMS@GOx, can be used for CDT/starvation therapy/PTT/immunotherapy for synergistic cancer treatment. The experimental results showed that CMS containing multivalent elements such as Cu^l+/2+^ and Mo^4+/6+^ possessed Fenton-like, GSH peroxidase and catalase activities. CMS produced ·OH through a Fenton-like reaction after entering the tumor, and this process would deplete GSH overexpressed in TME, thus reducing the antioxidant capacity of cancer. CMS with peroxidase activity can generate O_2_ by reacting with intracellular H_2_O_2_ under hypoxic TME conditions, activating GOx-catalyzed oxidation of glucose and achieving H_2_O_2_ regeneration. This regeneration could perform a Fenton-like reaction and enhance CDT efficacy. Meanwhile, CMS under 1064 nm laser irradiation exhibited excellent photothermal conversion efficiency (η = 63.3%) and produced large amounts of superoxide anions, showing effective tumor killing. In an *in vivo* anti-tumor assay targeting cervical cancer cells in U14-bearing mice, it was surprising to find that the synergistic treatment of PEGylated CMS@GOx combined with CTLA-4 antibody significantly enhanced CD4^+^T and CD8^+^T infiltration in tumors and reduced Tregs considerably. This mesopore-mediated anti-tumor strategy effectively ablated primary tumors and inhibited cancer metastasis by eliciting a robust immune response. It provides an innovative idea for synergistic integrated cancer therapy and is of significant value for future clinical translation.

**Figure 4:**
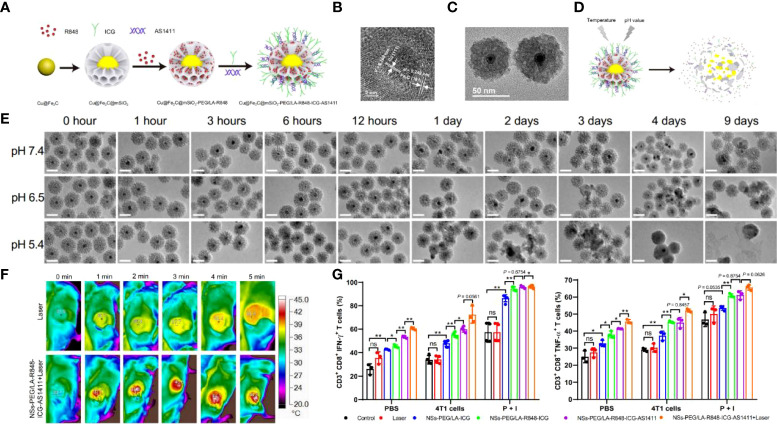
**(A)** Schematic diagram about the Cu@Fe_2_C@mSiO_2_-PEG/LA-R848-ICG-AS1411 nanozymes. **(B)** HRTEM images of Cu@Fe_2_C NPs. **(C)** TEM of Cu@Fe_2_C@mSiO_2_ NSs. **(D)** Schematic representation of degradation and drug release from temperature and pH-controlled Cu@Fe_2_C@mSiO_2_-PEG/LA-R848-ICG-AS1411 nanoenzymes. **(E)** TEM images of Cu@Fe_2_C@mSiO2 under different environments (pH=7.4, 6.5, 5.4) at different times. **(F)** Thermal infrared imaging of tumor-bearing mice after intravenous injection of PBS and Cu@Fe2C@mSiO2-PEG/LA-R848-ICG-AS1411 under 808 nm laser irradiation. **(G)** Flow cytometric statistical analysis of CD3^+^CD8^+^IFN-γ^+^ and CD3^+^CD8^+^TNF-α^+^ in mouse splenocytes after receiving different treatments (n = 3, ns, no significance, *p < 0.05, **p < 0.01 (87). Reprinted with permission from Ref. (87).

Iron-based nanomaterials are widely cited for antitumor therapy because of their excellent CDT properties in an acidic environment (84). Coincidentally, TME and intracellular lysosomes can provide acidic environment sites (85, 86). Hou et al. (87) designed a mesoporous-based visualization of the immunomodulatory nano-mimetic enzyme Cu@Fe_2_C@mSiO_2_-PEG/LA-R848-ICG-AS1411. Fe_2_C has good photothermal conversion properties and CDT agents, and copper ions enhance the effect even further. Also, Fe_2_C can provide real-time images under magnetic resonance imaging (MRI). As a nano delivery platform with excellent biocompatibility, the pore size of mSiO_2_ can be loaded with a large amount of immuno-agonist (R848) and pH/temperature-sensitive release material PEG/LA (88). Finally, NIR-II fluorescent imaging agent ICG and tumor-targeting aptamer AS1411 were modified on the nanomaterial surface **(** **Figure 4A** **)**. The results showed that the Cu core diameter of Cu@Fe_2_C was about 4 nm, the Fe_2_C shell thickness was about 20 nm, and the particle size reached 55 nm after encapsulation of mSiO_2_ **(** **Figures 4B, C** **)**. The modification of PEG/LA enables the nanomaterials to respond effectively to pH and temperature and improve the therapeutic effect by drug targeted release in acidic environments **(** **Figures 4D, E** **)**. With 4T1 and B16F10 tumor-bearing mice as experimental subjects, the nanosystems were found to reach maximum enrichment at the tumor sites in mice 24 hours after injection. Laser irradiation at this time revealed a rapid increase in tumor site temperature from 29.1°C to 44.8°C, while the laser-only control group increased from 31.9°C to 38.3°C **(** **Figure 4F** **)**. And the tumors of in situ, as well as metastatic tumor-bearing mice treated with this strategy, were significantly suppressed, and survival was very prolonged. When exploring the tumor suppression factors, it was found that Cu@Fe_2_C@mSiO_2_-PEG/LA-R848-ICG-AS1411 significantly upregulated CD80 and CD86 expression in peritumor DCs and lifted the TIME by downregulating the ratio of MDSCs. On the other hand, the nanomaterials also induced the proliferation and activation of CD8^+^T **(** **Figure 4G** **)**. This strategy not only accomplishes the purpose of multiple combination therapy of PTT/CDT/immunotherapy but also combines MRI/NIR-II double-mode imaging to guide tumor treatment. This mesoporous material-based synergistic therapeutic system breaks the bottleneck of single-modality tumor treatment with low efficiency, high toxic side effects and easy drug resistance, which is highly significant for optimizing cancer treatment protocols.

## Cell membrane-modified nanomaterials

As an integral part of cells, the cell membrane is a bilayer structure mainly composed of lipids, proteins and a small number of sugars, which exercises the functions of intercellular material exchange and information transfer. Due to the excellent biocompatibility, modifiability and low immunogenicity of cell membranes. Nanosystems constructed on this basis have vigorously promoted the development of tumor immunotherapy (89–91). By altering their physicochemical properties, biofilm nanoparticles allow for efficient drug loading and minimize the removal of nanoparticles once they enter the bloodstream. The therapeutic goal is reached through durable circulation *in vivo* and gentle release of the drug at the designated site (92, 93). This section reviews the applications of tumor cell membranes, immune cell membranes, and erythrocyte membrane-modified nanomaterials in tumor immunotherapy.

### Tumor cell membrane modification

Cytotoxic T lymphocytes (CTLs) perform anti-tumor functions by combining with receptors on tumor cell membranes and recognizing tumor cells (94, 95). Bionic materials constructed from cancer cell membranes (CCMs) are platforms with anti-tumor potential. Tumor cell membranes are readily available, thanks to the rapid proliferation of tumor cells, and their membrane proteins can be homologously targeted to tumors and have immune evasion capabilities. However, the effective utilization of CCMs still faces problems such as unstable expression of surface tumor antigens or insufficient tumor-targeting ability. Existing studies have been carried out by functionalized modification of CCMs to obtain enhanced targeting or activation of the anti-tumor immune response (96).

A typical nano-vaccine consists of an antigen (e.g., protein, peptide, DNA, etc.), an adjuvant and a vector to trigger specific anti-tumor immunity (97, 98). However, the expression profile of tumor antigens varies considerably between individuals due to the tumor patient heterogeneity. The use of tumor cell-derived membrane-structured encapsulated nanoparticles (NPs) allows a valuable solution to these problems. Liu et al. (99) used PLGA-modified membrane-loaded R837 from B16-OVA cancer cells and finally modified it with mannose. The results showed that the obtained nano-vaccine NP-R@M-M was effectively taken up by APCs and stimulated the maturation of these cells, thus triggering an anti-tumor immune response. *In vivo* experiments, NP-R@M-M effectively migrated to draining lymph nodes (LNs) and initiated immunity after intradermal injection. Notably, tumor progression was effectively inhibited in the mice treated with NP-R@M-M nano-vaccine immunization in combination with anti-PD-1. Half of the mice were tumor growth-free and achieved the most prolonged disease-free survival. The tumor cell membrane-encapsulated adjuvant nanoparticles circumvent the significant inter-individual variation in tumor antigen expression profiles and the possible failure of tumor lysates to trigger anti-tumor immune responses. Therefore, it will be interesting to use tumor cell membranes as tumor-specific antigens to develop cancer vaccines.

Mature DCs act as APCs to kill tumor cells by initiating specific T cells (100). Tumor vaccines against a single antigenic target are challenging to alleviate clinically relevant symptoms in cancer patients. Biofilm vaccines provide a new idea to solve the above problems but also face the problem that some tumor antigens are expressed intracellularly but not on the membrane. In response, Zhang et al. (101) proposed using bioreprogrammed cell membrane NP@FM of DCs and cancer cell-derived fusion cells (FCs) as a tumor vaccine and fusing two types of immune-associated cells for robust expression of the whole tumor antigen complex and immune co-stimulatory molecules. For better tracing, MOF@FM was obtained by labeling with a fluorescent metal-organic framework (MOF). Results showed that the percentage of tumor-free mice was 60% in MOF@FM within 36 days, superior to the therapeutic effect of DCs alone or tumor cell membrane-encapsulated nanomaterials. MOF@FM induced a significant secretion of IFN-γ and IL-6, and CD3^+^CD8^+^CTLS, suggesting that it was expected to enhance anti-tumor immunity. Its effect on immune-critical cells DCs have also been demonstrated (102). NP@FM can function as APCs for T-cell immune activation, and DCs can recognize tumor antigens carrying NP@FM to induce DCs-mediated T-cell immune activation. Combining these two immune activation pathways provides a potent anti-tumor immune response. Simultaneously mimicking APCs and cancer cells, this cell membrane vaccine strategy allows for developing multiple vaccines against multiple tumor types and provides opportunities for tumor immunotherapy.

Bacterial-derived substances can enhance specific anti-tumor responses by promoting adaptive immune responses. Conversely, they are unable to cause an immune memory response, nor do they provide long-term protection against tumors (103). Also, tumor cell membranes have been used to develop tumor vaccines. Still, they are limited by their inability to produce long-term therapeutic effects due to factors such as the weak immunogenicity of membrane antigens. Although based on these problems, Nie et al. (104) designed a combination vaccine incorporating autologous tumor membrane (TM) antigen and bacterial cytoplasmic membrane adjuvant for enhanced anti-tumor immune response. The study mixed E. coli cell membranes and autologous-derived tumor cell membrane antigen into nanoparticles to form hybrid membrane nanoparticle vaccine HM@NPs. The results showed that HM@NPs effectively induced tumor regression in mice with *in vivo* tumor models of colon adenocarcinoma cells CT26, triple-negative breast cancer cells 4T1, melanoma cells B16-F10, and triple-negative breast cancer cells EMT6. Notably, the hybrid membrane nanoparticle vaccine elicited strong tumor-specific immune memory protection for up to 3 months, which prolonged postoperative survival in mice and effectively prevented tumor recurrence. The study did not find that E. coli cell membranes overstimulated the immune system and caused a factor storm. Based on the combined application of bacterial cytoplasmic and tumor cell membranes, this vaccine could efficiently trigger the innate immune. It is of great significance for individualized tumor immunotherapy and the prevention of postoperative recurrence using autologous tumor membrane extraction.

### Immune cell membrane modification

As a significant component of the TME, TAM fuels tumors in multiple ways, leading to immunosuppression and resisting therapy (105, 106). TAM domestication is becoming a feasible option for treating tumors. CSF1 is a critical regulatory messenger of macrophages and maintains the tumor promotion function of TAM. Blocking CSF1-CSFIR could relieve the immunosuppression of TME and thus improve the efficacy of immunotherapy. Feng et al. (107) obtained NPR@TAMM using TAM membranes (TAMM) with homing ability and good immunocompatibility overlaid onto upconverted nanoparticles containing photosensitizers. The results showed that NPR@TAMM depleted CSF1 of TME and simultaneously blocked the interaction of TAM with cancer cells. PDT combined immunotherapy-mediated NPR@TAMM polarized macrophages to M1 phenotype and induced ICD. This process also stimulated the production of effector T cells by activating APCs. Primary and distant tumor models were constructed and treated in mice. The NPR@MM+NIR group significantly suppressed primary and metastatic tumors, considered a result of immune system activation. Such agents, represented by antibodies or small molecules, are challenging to apply because of their low response rate. TAMs membrane-coated modified delivery systems avoid nanoparticle clearance by the reticuloendothelial system, thereby driving nanoparticle accumulation in the TME. At the same time, it inhibits tumor growth by competitively inhibiting the interaction of TAM with metastasis and, crucially, reducing serum CSFI levels and blocking the CSFI/CSFIR signaling pathway, leading to immunosuppressive inactivation. These advantages of TAMM can be exploited in combination with other types of therapy to explore new options for tumor immunotherapy.

**Figure 5:**
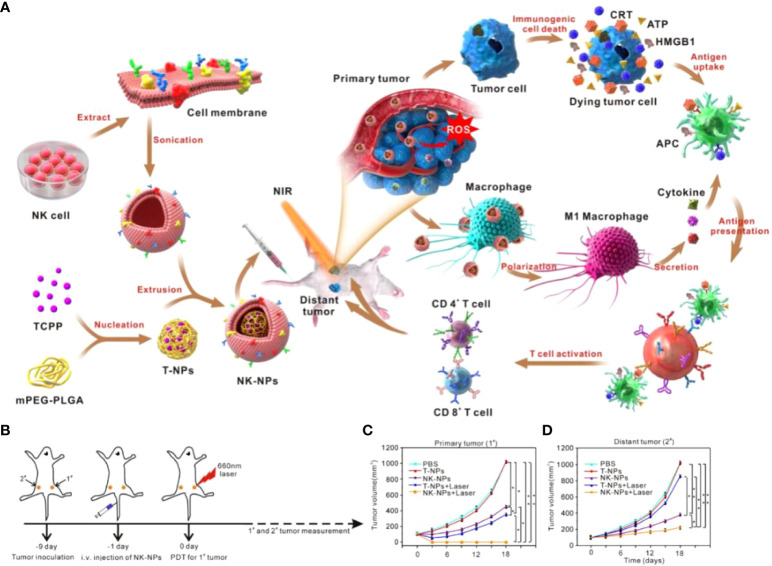
**(A)**. Schematic diagram of NK-NPs encapsulated in NK cell membranes to suppress tumors with immunotherapy. **(B)** Schematic diagram of the treatment. The tumor on the right is defined as a “primary tumor” and treated with PDT, whereas the tumor on the left is designated as a “distant tumor” and not treated with PDT. **(C)** Growth curves for the primary tumors. **(D)** Growth curves for the distal tumors (112). Reprinted with permission from Ref. (112). Copyright© 2018, copyright Deng et al.

Natural killer (NK) cells are unique and vital among immune cells, serving as gateways against infection and cancer, and have received significant attention in tumor immunotherapy research (108, 109). Activated NK cells can directly kill target cells and indirectly suppress cancer by secreting inflammatory and chemokines by releasing two molecules, perforin and granzyme (110, 111). Cai et al. (112) prepared TCPP-loaded NK cell membrane-encapsulated PLGA nanoparticles (NK-NPs). NK-NPs can precisely target tumors to achieve treatment by 1. directly killing primary tumors through PDT therapy; 2. inducing activation of APCs by dead tumor cells; 3. inducing polarization of M1 macrophages **(** **Figure 5A** **)**. NK-NPs and T-NPs-treated cells produced large amounts of ROS under NIR irradiation. And the former was about 5 times more than the latter, which was due to the specific tumor-targeting ability of NK-NPs. Treatment of tumor-bearing mice with NK-NPs + NIR irradiation showed significant inhibition of primary and distant tumors and the highest survival rate **(** **Figures 5B-D** **)**. This was because NK cells contributed to the maturation of APCs, thereby activating T-cell killing capacity. Importantly, NK-NPs induced macrophages to polarize toward the M1 phenotype. Therefore, using NK cell membranes combined with PDT is a non-invasive treatment option that can directly generate ROS to kill tumors. And it promotes T cell production and proliferation by triggering M1 macrophage polarization and stimulating the immune system to achieve highly effective tumor immunotherapy.

Myeloid-derived suppressor cells (MDSCs) facilitate tumor immune evasion in various ways, including the suppression of T-lymphocyte activity. Wang et al. (113) prepared neutrophil membrane-mimetic nanoparticles (pCSs) with neutrophil membranes by wrapping neutrophil membrane vesicles (NMVs) around PLGA nanomaterials. It showed that in addition to resembling polymorphonuclear MDSCs (PMN-MDSC), pCSs inherited most of the membrane receptors of neutrophil membranes. More importantly, pCSs could suppress the extension of MDSCs and tumor translocation. In a murine melanoma homozygous model, injection of pCSs resulted in reduced MDSCs aggregation in the tumor and surrounding lymphoid organs without inducing compensatory inward flow of any alternative myeloid subpopulation. Significantly, pCS reinstated the function of T lymphocytes, thereby considerably reducing the progression of tumor-bearing mice. Furthermore, pCS could cooperatively suppress tumor advancement and prolong survival when used in combination with PD-1. This study avoided a simple removal of MDSCs that could result in serious adverse outcomes. It provided a secure and practical approach to cancer therapy by inhibiting the expansion of MDSCs and their transport to the tumor.

### Erythrocyte membrane modification

The biconcave morphology of erythrocytes gives them excellent deformability and allows for smooth passage through capillaries of several microns. Importantly, erythrocytes are considered an effective bionic drug carrier due to their long circulation time and easy availability in the body (114).

Porphyromonas gingivalis (Pg) is an anaerobic Gram-negative bacterium that stably secretes melanin during gingival and periodontal infections and significantly promotes macrophage polarization toward the M1 phenotype. Zhou et al. (115) designed an erythrocyte film coating Porphyromonas gingivalis (cmPg). The results showed that the coating both improved the size distribution of bacteria and reduced the clearance of bacteria by macrophages. Polarization of bacteria on macrophages was also achieved. CmPg was able to stably produce melanin, which can be used as a photothermal therapeutic agent of PTT under laser irradiation to promote the occurrence of photothermal induced ICD in cancer cells. This process activates the immune system and stimulates the increase of DCs maturation, cytotoxic lymphocytes, memory T cells and anti-tumor M1 macrophages. This combination of immunomodulatory bacteria and PD-1 antibody suppressed both primary and secondary tumors. The study provides a new idea for tumor immunotherapy by transforming inflammatory bacteria such as Pg into biomaterials for cancer prevention and combining them with other therapeutic options, with an uncomplicated production process and a precise mechanism that facilitates clinical translational studies at a later stage.

To protect the organism, senescent or damaged red blood cells are often removed by macrophages and DCs such as those found in the spleen. As a critical lymphoid organ, the spleen is rich in immune cells that can participate in anti-tumor immune regulation. Inspired by this, damaged erythrocytes were hypothesized to be used to enrich lymphoid organs to deliver TAAs to APCs. According to this idea, Wang et al. (116) fused tumor cell membrane-associated antigens with nano-erythrocytes by ultrasound and membrane extrusion and loaded tumor antigens onto nano-erythrocytes to obtain nano-Ag@erythrosomes containing tumor antigens. Results showed that nano-Ag@erythrosomes could accumulate in the spleen within 1 hour after intravenous injection. DCs and macrophages up-regulated the expression of immune-related markers in mice’s spleen after stimulation. Cell populations internalizing nano-Ag@erythrosome showed much stronger stimulation markers than un-uptaken DCs and macrophages. Meanwhile, PD-L1, a suppressor molecule on DCs and macrophages, expression was also increased after nano-Ag@erythrosome, suggesting that nano-Ag@erythrosomes combined with PD-L1 blockade of splenic APCs could induce potential synergistic anti-tumor effects. Notably, splenic NK cells, B cells, CD4^+^T cells and CD8^+^T cells were also activated by nano-Ag@erythrosomes, and PD-L1 antibodies could amplify their induced anti-cancer immune responses. A customized cancer vaccine is critical for patients because each patient’s genetic mutation or tumor progression may result in distinctive tumor antigens. It is believed that with continuous in-depth exploration and research, cell membrane nanosystem-based tumor immunotherapy will achieve more remarkable breakthroughs and broader applications, promoting the development of human anti-tumor research.

The presence of blood-brain-barrier (BBB) makes the treatment of Glioblastoma (GBM) unsatisfactory. It hinders drug delivery efficiency and results in low local drug concentrations in the tumor, while systemic high-dose drug delivery is bound to have serious side effects (117). In addition, TIME is also a critical barrier to GBM treatment failure. Microglia and macrophages account for more than 30% of the tumor cell mass, so a regimen with efficient BBB penetration and effective TIME reversal is essential for GBM treatment (118, 119). Notably, both microglia and macrophages can differentiate into M1 or M2 types under stimulation. Since they undertake functions such as phagocytosis and antigen presentation, the focus of GBM immunotherapy is not to reduce their numbers but to re-educate their phenotype. It was found that miR-155 can regulate M2 to M1 polarization (120). Zhang et al. (121) designed and constructed an erythrocyte membrane-encapsulated nucleic acid nanogel (Vir-Gel) in which nucleic acids were modified by functional peptides that mimic viral structures. The erythrocyte membrane surface was modified using M2pep peptide, which targets M2 phenotype cells, and HA2 peptide of influenza virus origin, which promotes the fusion of the erythrocyte membrane and endosomal membrane and ensures efficient delivery of miR155. *In vivo* experiments showed that Vir-Gel led to a substantial increase in survival time in tumor-bearing mice compared to controls, indicating that this strategy is expected to be a windfall for GBM treatment.

## Metallic materials in tumor immunization

As an essential component of living organisms, metal elements and metal-related proteins are involved in nearly all life processes, including signal transduction, energy transfer, redox regulation, and molecular synthesis and degradation. Metals are also involved in immune regulation, including immune sensing and defense. Importantly, metal ions can address the failure of immunotherapy due to inadequate immune activation, and this option for immune response and tumor treatment strategies is gradually coming into the limelight. This section will review the application of metal particles, metal ions and MOF in tumor immunotherapy.

### Metal particles

Iron oxide nanoparticles have a promising clinical future because of their properties like superparamagnetic and catalytic activity. Ferumoxytol has received FDA approval for the treatment of iron deficiency, and it was an ultra-small iron oxide nanoparticle with a hydrophilic carboxymethyl-dextrose coating. Daldrup-Link et al. (122) used ferumoxytol to validate its role in tumor treatment. The results indicated that ferumoxytol significantly inhibited tumors and confirmed the reduction of liver tumors in treated mice through a small cell lung cancer hormonal mouse model. Ferumoxytol significantly increased caspase-3 levels to inhibit tumor growth and increased TNF-α and IL-10 levels in macrophages, inducing M1 macrophage polarization and thus inhibiting tumor spread. This experiment minimizes systemic toxicity by using FDA-approved Fe_3_O_4_-containing coated nanoparticles alone. The TME has been a significant challenge for tumor immunotherapy. Using bio-nano materials as carriers for targeted delivery of relevant drugs into tumors has been one of the hot spots in tumor immunotherapy. This study found that the non-drug-carrying Fe_3_O_4_ nanoparticles also activate suitable immune cells, which may be developed as novel tumor immunotherapy.

As nanoparticles are increasingly used in the medical field, their safety and effects on the human immune system are gradually being emphasized. Gold-core nanoparticles (GNPs) are commonly used in medical areas because of their optical properties and high biocompatibility. GNPs can induce an effective immune response at low doses (123). B-lymphocytes are responsible for antibody production in the immune response and are an essential component of the body’s immune system. Therefore targeting B lymphocytes becomes significant for developing prophylactic and therapeutic vaccines. Clift et al. (124) To verify the effect of GNPs on the immune function of B lymphocytes, GNPs with different surface modifications PEG, PEG/PVAand shape (spherical, rod-shaped) characteristics were co-incubated with newly isolated human B lymphocytes. The results showed that the polymer-coated GNPs interacted less well with B lymphocytes than uncoated GNPs. Noteworthy. None of the above GNPs significantly affected cell viability, i.e., by incubation for 24 h using a concentration of 20 ug/mL. Except for GNPs with rod-like structures and spherical GNPs without polymer coating, none of the GNPs affected the activation of markers in nascent B lymphocytes or increased secretion of inflammatory substances. This study on the effects of different modifications and morphologies of gold nanoparticles on B-lymphocytes provides a good direction for drug delivery targeting B-lymphocytes. It is possible to achieve targeted drug delivery and immunostimulation protocols while minimizing side effects.

### Metal ions

Chemotherapy and CDT can induce anti-tumor immunity by inducing ICD in tumor cells. However, conventional small molecule drugs have limitations, such as high toxicity to normal tissues and rapid clearance from reticuloendothelial system organs. Designing a nanoplatform that can be used for CDT with effective loading and targeted release of chemotherapeutic drugs is crucial. Shi et al. (125) constructed DOX-TAF@FN using a metal-phenol network loaded with the chemotherapeutic drug Dox formed by the coordination of polyphenols and metal ions. Among them, TAF nanocomplexes are formed by chelating Fe^3+^ with polyphenol tannic acid (TA), which have excellent biosafety and biocompatibility, and are also stable pH-responsive. FN is fibronectin that targets cancer cells with high expression of α_v_β_3_ integrin. TA can convert Fe^3+^ to Fe^2+^ after TAF dissociation in TME. Fe^2+^ reacts with hydrogen peroxide of glycoside content in tumor cells by Fenton reaction to produce cytotoxic hydroxyl radicals and intracellular GSH, enhancing the induction of cancer cell ferroptosis. In addition, the iron ions dissociated by TAF nanocomplexs under TME have an r1 relaxation rate, enabling T1-weighted MR imaging. The results show that the nanoplatform has good MR imaging, aggregates at the tumor site, and can be metabolized by the reticuloendothelial system. Therapeutically DOX-TAF@FN synergized with PD-L1 antibody, CD4^+^T, CD8^+^T, and NK cell expression at tumor sites were upregulated, and Tregs were significantly downregulated, allowing for potent tumor suppression. Studies have shown solid immune death based on Fe-generated CDT and successful activation of DOX chemotherapy. It also effectively alleviated the TIME at the tumor site, prevented immune evasion, and conclusively enhanced the anti-tumor immune system *in vivo*.

### MOFs

Since MOFs were proposed to have good loading capacity and controlled release advantages, they have been widely used in drug delivery and clinical oncology therapy. In contrast to conventional drug carriers, these porous coordination polymers (PCPs) consist of metal ions or clusters covalently bound to organic linkers (126). Many advantages are offered: versatile modifiability, stimulation of reactive drug controlled release, high loading capacity, and biodegradability ensured by weaker ligand bonds (127).

**Figure 6:**
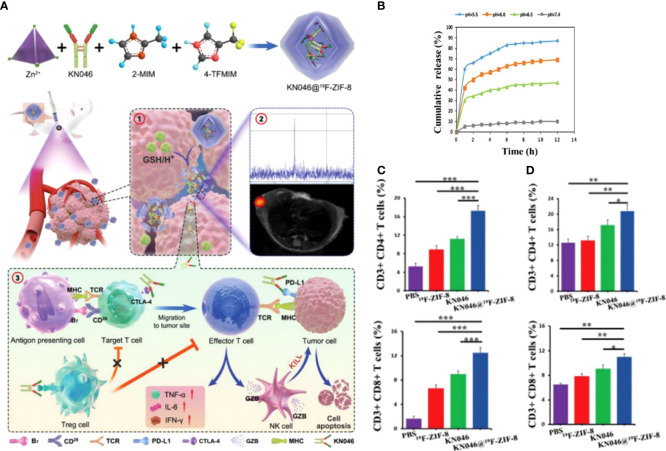
**(A)**. Schematic diagram of the structural composition of KN046@19^F^-ZIF-8 nanoplatform and ① Schematic diagram of KN046@19^F^-ZIF-8 in response to tumor microenvironment and dissolution of release. ② 19^F^ NMR signals response to GSH/H^+^. ③KN046@19^F^-ZIF-8 combined with dual-targeted immune checkpoint inhibitors for anti-tumor immunotherapy. **(B)** cumulative release of KN046 from KN046@19^F^-ZIF-8 in PBS at different pH (pH =5.0, 6.0, 6.5, 7.4). **(C, D)** The number of CD4^+^T and CD8^+^T cells in the tumor and spleen was measured and quantified by flow cytometry after treatment with different treatment regimens (PBS, 19^F^-ZIF-8, KN046, KN046@19^F^-ZIF-8) for the tumor-bearing mice. Data are expressed as mean ± SD (n = 5, *p < 0.05, **p < 0.01,***p < 0.001) (128). Reprinted with permission from Ref. (128).

Immune checkpoint inhibitors with CTLA-4 and PD-L1 antibodies are effective immunotherapeutic agents, but the response rate of single-agent immunotherapy limits the clinical benefit. Dual-antibody combination immunotherapy can effectively improve immunotherapeutic efficacy but suffers from high price and side effects. KN046 is a reconstituted PD-L1/CTLA-4 bispecific single-domain antibody Fc fusion protein that explicitly binds PD-L1 and CTLA-4 and effectively kills tumor cells by blocking the combination of PD-L1 with PD-1 and CTLA-4 with CD80. To get rid of the off-target effect brought and deliver KN046 to tumors safely and effectively, Song et al. (128) used a non-toxic degradable material, ZIF-8, to encapsulate KN046 to obtain KN046@ZIF-8. ZIF-8, a metal-organic backbone based on zinc (Zn^2+^), could be rapidly degraded in tumors by highly expressed and weakly acidic GSH **(** **Figures 6A, B** **)**. 19^F^-MRI probe was introduced to monitor the antibody release in real-time, and its signal could be initiated when ZIF-8 is disrupted. The results showed that the KN046 antibody was released in tumor sites but not in normal tissues, which effectively enhanced the killing of tumor cells by blocking the combination of PD-L1 with PD-1 and CTLA-4 with CD80/CD86. In animal experiments, the percentages of CD3^+^CD4^+^T and CD3^+^CD8^+^T cells in the tumor **(** **Figure 6C** **)** and spleen **(** **Figure 6D** **)** of the KN046@19^F^-ZIF-8 group were dramatically enhanced. On the contrary, the number of Tregs was reduced considerably. Serum TNF-α, IL-6 and IFN-γ levels increased markedly after receiving the KN046@19^F^-ZIF-8 injection. Mice ultimately treated with KN046@19^F^-ZIF-8 exhibited the minimum relative tumor volume and the maximum survival rate. As an intelligent nano-delivery vehicle, the bio-responsive MOF material also exhibited excellent biocompatibility and biodegradable properties. It protects the active components of immunotherapy from damage and allows accurate delivery of antibodies to the tumor region.

MOFs have attracted significant interest in oncology therapy because of their excellent programmability and drug loading properties. Most MOFs involve metal ions and organic molecules with multi-dentate structures connected by coordination. In contrast, MXFs are framework materials that introduce metal ions and organic molecules as ligand units. DNA can form polycrystalline MXFs in concert with various metal ions due to its superior programmability and biocompatibility. Tan et al. (129) first synthesized Hf-CpG, Zn-CpG and Ca-CpG to demonstrate that the metal ion-DNA coordination strategy is a versatile method for synthesizing MXFs. Their sizes are around tens to one hundred nanometers and have stable crystal structures. For example, Hf-CpG MXFs containing high Z-element Hf are synthesized. The structure can concentrate more X-ray radiation dose into the tumor, inducing damage to the primary tumor. Meanwhile, tumor-associated antigens produced by radiotherapy and CpG-based MXFs induce a systemic solid anti-tumor immune response. This radiotherapy strategy based on the structure of Hf-CpG MXFs inhibited the growth of distant metastases and prolonged immune memory effect to prevent a recurrence. *In vivo* experiments have shown that external beam radiation therapy to tumors locally injected with Hf-CpG MXFs can eliminate the primary tumor, inhibit tumor metastasis, and protect cancer from tumor re-attack by stimulating a potent immune response. The study offered directions for manufacturing various rationally designed MXFs with desired functions and suggested a strategy to stimulate systemic immune responses through local radiotherapy only.

## Hydrogel materials for combinatorial immunotherapy

There has been considerable interest in drug delivery schemes using biomaterials as carriers to achieve local tumor suppression and control systemic anti-tumor immune responses with reduced systemic toxicity by delivering to tumor sites or postoperative tumor areas. Due to the particular network-like structure, hydrogels have high drug loading efficiency. They can determine their release depending on the modified composition and the mesh size, making them often designed as platforms for targeted drug delivery. This section reviews hydrogel-based immunotherapeutic materials through three different administration modes: local injection, local non-injection, and oral delivery.

### Local injection of hydrogel

S-nitrosoglutathione (GSNO) is a common NO donor used in biological research, leading to local and systemic expansion and activation of DCs. Still, its function is inhibited by the expansion of other immune cells expressing CTLA-4. Thomas et al. (130) synthesized a thermosensitive hydrogel F127-*g*-using Pluronic^®^ F127 and gelatin by a simple chemical coupling Gelatin hydrogel loaded with NO donor GSNO and CTLA-4 antibody. The results showed that the F127-*g*-gelatin hydrogel combined with GSNO and CTLA-4 antibody had more substantial anti-tumor effects on tumor-bearing mice than saline, bare hydrogel and free GSNO + CTLA-4 antibody. It demonstrated the synergistic therapeutic effect of GSNO combined with CTLA-4 antibody. It showed the potential of GSNO and CTLA-4 antibody combinations to treat different tissue sites and the potential biology of the local area retardation platform. This injectable temperature-sensitive hydrogel relies on its gel formation and degradation properties. It facilitated lymphatic uptake by maintaining drug retention in tumors, triggered release by responding to TME and formed micelles suitable for lymphatic absorption. It provides a controlled release system that is simple to synthesize, can be loaded with various drug types, does not require surgery, and circumvents side effects such as blood pressure changes associated with NO systemic administration. Local release systems can improve cancer immunotherapy’s local and distant products while minimizing repeat dosing and addressing the obstacles of poor patient adherence.

**Figure 7:**
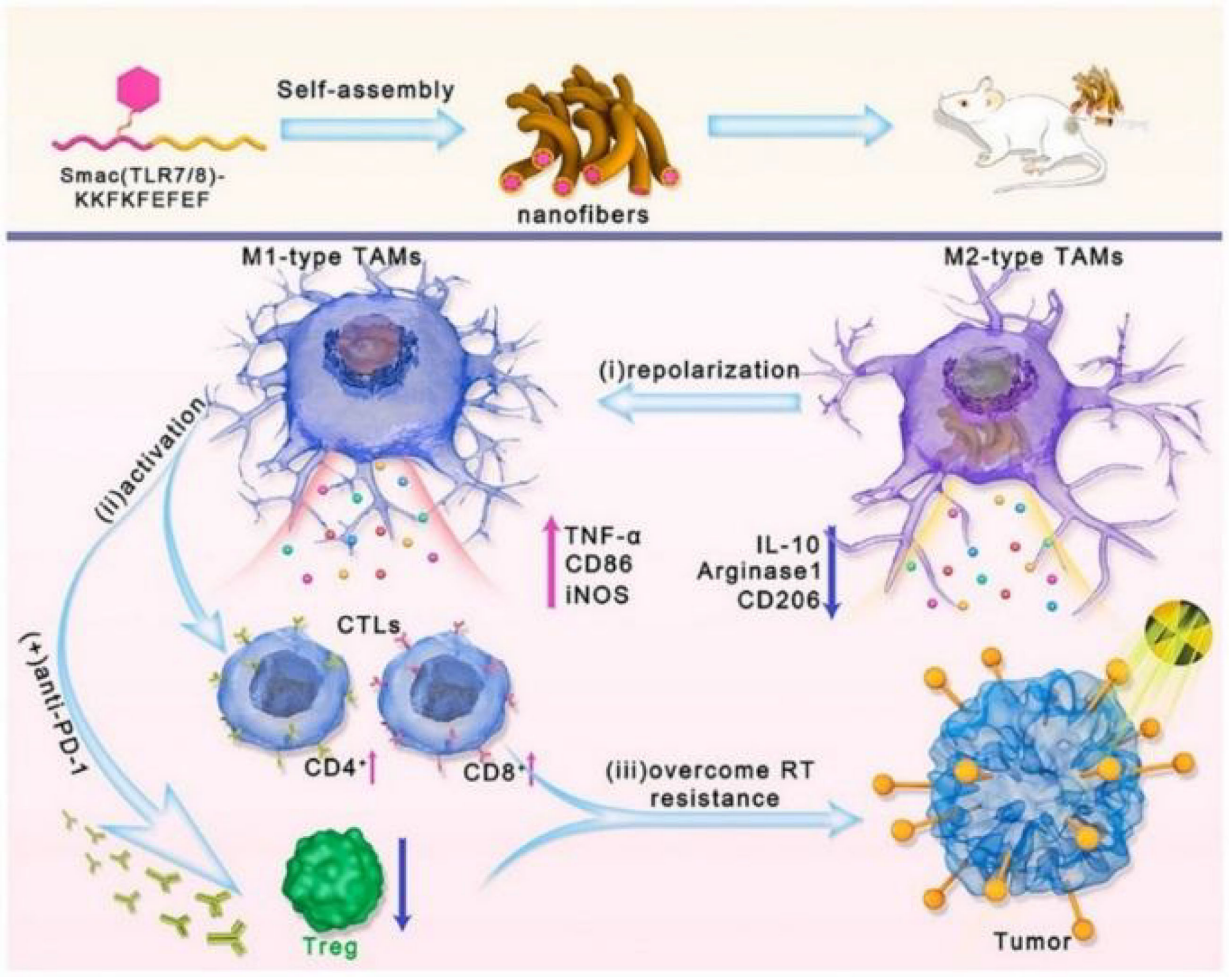
Schematic of the Smac-TLR7/8 hydrogel regulates macrophage repolarization to overcome radioresistance (133). Reprinted with permission from Ref. (133).

Radiotherapy (RT) promotes tumor cell apoptosis through high-energy radiation as a standard treatment option for solid tumors. However, the presence of TIME dramatically reduces the efficacy of RT, and as a significant component of TIME, M2 was found to be a typical RT-tolerant cell (131). Moreover, when tumor patients receive RT, tumor cells highly express inhibitors of apoptosis proteins (IAPs), blunting tumor sensitivity to RT. At the same time, the increase in the number of M2 phenotypes synergistically accelerates the process of RT failure (132). Therefore, reconstitution of TIME by re-education of M2 may be optional to overcome RT resistance. Based on the above background, Liu et al. (133) could self-assemble Smac-TLR7/8 peptides forming a nanofibrous hydrogel structure. The Smac peptide fragment disengages its apoptosis-inhibiting function by binding to IAPs, while the TLR7/8 agonist polarizes M2 to M1 phenotype, synergistically restoring tumor radiosensitivity (134). Repolarized M1 enhances the immune response by recruiting TILs and weakening Treg cells **(** **Figure 7** **)**. Animal experiments using B16 tumor-bearing mice administered by peritumoral injection revealed that Smac-TLR7/8 hydrogels formed by *in situ* self-assembly, effectively prolonging the retention time of TLR7/8. In the absence of RT, tumor suppression reached 50.3%, while in combination with RT, this figure increased to 57.2% and reached 86.3% within 14 days. Analysis of treated tumor tissues revealed a remarkable M2 to M1 polarization after treatment with Smac-TLR7/8 hydrogel + RT and a substantial rise in CD4^+^T and CD8^+^T due to the lifting of TIME. Furthermore, combined with immune checkpoint blockade therapy, Smac-TLR7/8 + RT achieved excellent results. Although conventional treatment protocols similar to RT have been better developed, they still hinder treatment progress due to phenomena such as resistance. This Smac-TLR7/8 hydrogel strategy brings new hope to tumor treatment by remodeling TIME while addressing opposition to RT.

### Local non-injection of hydrogel

**Figure 8:**
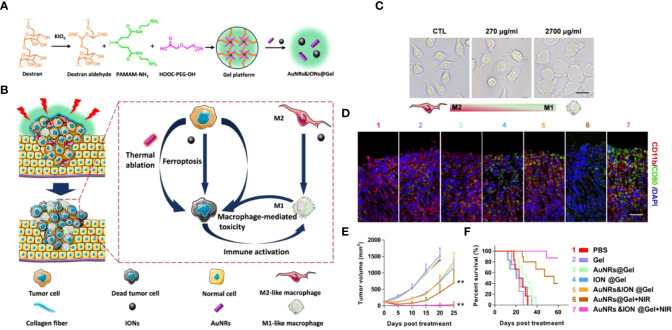
**(A)**. Schematic diagram of AuNRs&IONs@Gel synthesis. **(B)** Schematic diagram of AuNRs&IONs@Gel induced tumor cell death that can be used for PTT, ferroptosis, and immunotherapy. **(C)** Morphological images of M2-type macrophages after co-incubation with IONs (Scale Bar = 10 μm). **(D)** Immunofluorescence staining of tumor sections from mice receiving different treatments, CD11b (red) and CD80 (green) (Scale Bar = 10 μm). **(E)** Tumor size of receiving different treatment modes after 25 days (n = 5). **(F)** Survival curves of mice treated with different regimens (n = 10). Treatment groups: 1, PBS; 2, Gel; 3, AuNRs@Gel; 4, IONs@Gel; 5, AuNRs&IONs@Gel; 6, AuNRs@Gel + NIR; 7, AuNRs&IONs@Gel + NIR (**p < 0.01) (136). Reprinted with permission from Ref. (136). Copyright© 2020, copyright Guo et al.

With the rapid development of nanomedicine in biological applications, its interaction with biological systems (e.g., stability and toxicity profile in the blood) has received increasing attention. It has been found that the vast majority of intravenously administered nanomedicines are eliminated by the mononuclear phagocytic system (135). For example, nanomedicines caught by Kupfer cells reside and are retained in the liver, increasing the risk of both inflammation and toxicity. Xu et al. (136) designed an AuNRs&IONs@Gel system consisting of gold nanorods (AuNRs) and iron oxide nanoparticles (ions) **(** **Figure 8A** **)**. The cavity structure possessed by the bladder offers the possibility of *in situ* tumor delivery, and the dextrose aldehyde on AuNRs&IONs@Gel can selectively adhere to bladder cancer collagen. This triple therapy performs tumor treatment by the following points: AuNRs perform photothermal treatment under NIR irradiation. A high local concentration of iron ions induces cancer cell ferroptosis, simultaneously polarizing M2 in TAM to an anti-tumor Ml-like phenotype. It exerts a direct anti-tumor effect and restores the tumor immune environment **(** **Figure 8B** **)**. The results showed that IONs induced RAW264.7 macrophage polarization from M2 to M1 with a dose-dependent approach *in vitro* **(** **Figure 8C** **)**. Immunofluorescence staining analysis of tumor tissues from the treated mice showed a decrease in CD11b (M2) cells and an increase in CD80+ (M1) cells (**Figure 8D**). Thetumor volume in the MB49 bladder cancer mouse model treated with AuNRs&IONs@Gel + NIR was almost undetectable within 20 days (**Figure 8E**). And the generation period has been significantly prolonged (**Figure 8F**). And DHE staining in tumor tissues showed that the gel platform resulted in a substantial increase in ROS in tumor cells. This hydrogel-based non-invasive drug delivery modality makes perfect use of the unique structure of the bladder and has transgenerational implications for bladder cancer treatment. Especially for patients who are intolerant to bladder surgery, this strategy will receive increasing attention for its excellent efficacy and safety.

Surgery remains the treatment for most patients with solid tumors at this stage. Unfortunately, due to tumor recurrence and metastasis, long-term survival rates for cancer patients have struggled to improve significantly. CAR-T therapies developed in this context have addressed certain malignant hematologic tumors to some extent, but their application in solid tumors remains challenging. Gu et al. (137) used acrylate-based modified HA as raw material with a cross-linking agent, loaded with CAR-T cells capable of targeting CSPG4 antigen (melanoma-specific antigen) and anti-PD-L1 blocking antibody-bound human platelets (P-αPDL1). Then it was loaded with PLGA nanoparticles packed with IL-15 to support CAR-T activity and proliferative capacity to obtain CAR-T-P-αPDL1@gel. Post-surgical inflammation triggered platelet activation, forming platelet-derived particles that released αPD-L1 antibodies to block PD-L1. Results showed that CAR-T-P-αPDL1@gel had the best anti-tumor effect, followed by CAR-T@gel combined with P-αPDL1 treatment, both of which were superior to free CAR-T cell therapy. And due to the long-term presence of CAR-T cells, suppression of contralateral tumor growth was observed in a double tumor model. This strategy takes advantage of the favorable chemokine environment in the tumor after surgical resection combined with CAR-T therapy to develop a degradable hydrogel repository. Storing CAR-T cells and P-αPDL1 removes residual tumor cells after surgery and prevents tumor recurrence.

### Oral hydrogel

Oral drugs face two fundamental problems: how to prevent degradation of the drug by enzymes and the acidic-base environment in the digestive tract and how to allow large molecules of biological drugs to penetrate the intestinal epithelium.

Gao et al. (138) screened the parent peptide H12 by phage display technology and subsequently optimized it. They obtained D-peptide A10Y (OPBP-1) with high PD-1/PD-L1 blocking activity and resistance to enzymatic degradation in the gastrointestinal environment. To facilitate its intestinal absorption, OPBP-1 was encapsulated in trimethyl chitosan (TMC) hydrogel to obtain OPBP-1@TMC. *In vivo* pharmacokinetic results in rats showed that the oral bioavailability of OPBP-1 exceeded 50%, with an oral half-life of 14.55 h. The tumor suppression found in CT26 colorectal carcinoma-bearing mice administered orally at 10 mg/kg was equal to that of the PD-L1 antibody. Compared with the control group, the percentage of CD8^+^T cells and the level of secreted IFN-γ were obviously enhanced in mice tumor tissues by OPBP-1@TMC treatment. OPBP-1 peptide-loaded with oral TMC hydrogel has superior bioavailability and promising anti-tumor efficacy with minimal side effects. Such protocols for immunotherapy delivered orally serve as an encouragement to promote further preclinical and clinical studies.

In addition to their role in the field of antitumor, the synthesis methods of nanomaterials are also worth discussing and can be simply divided into bottom-up and top-down. Bottom-up can be understood as a “small to large” process. For example, peptides can be formed into nano-hydrogels, nanofibers, nanotubes, etc. by self-assembly (139). Ouyang et al. (140) chemically modified the surface of the enzyme by a bottom-up strategy and encapsulated it in a porous framework. In contrast, top-down is a “large-to-small” process involving media milling and high-pressure homogenization schemes (141). Zhang et al. (142) prepared ultrathin two-dimensional boron nanosheets with multimodal tumor diagnostic and therapeutic integration using a new top-down technique. Interestingly, some nanomaterials can be prepared not only by bottom-up but also by top-down methods, depending on many factors, such as ease of preparation, efficiency and yield.

In addition to the abovementioned nanomaterials, many other nanomaterials modulate tumor immunotherapy through different modifications, assemblies, and so on. Of course, while we emphasize the moldability of nanomaterials, we should not ignore the impact of their properties, including nanometer size and shape, on immunotherapy. Take black phosphorus (BP) as an example. It has good near-infrared light absorption properties without any modification and is widely used in PTT, and the most representative ones are black phosphorus quantum dots (BPQDs) and 2D BP sheets (143–146). As an emerging inorganic nanomaterial, BP is widely used in the biomedical field due to its high biocompatibility and degradability. The phosphate produced by the reaction with oxygen in an aqueous solution is non-toxic to humans (147–149). In breast cancer staging, the absence or low expression of estrogen receptor (ER), human epidermal growth factor receptor 2 (HER2) and progesterone receptor (PR) is considered a triple-negative phenotype with a poor prognosis (150). Triple-negative breast cancer is ineffective against endocrine as well as HER2 therapy. Even though activation of the immune system is considered a practical approach against triple-negative breast cancer, there is still a high recurrence rate after single immune checkpoint therapy (151, 152). Zhang et al. (153) obtained nano-vesicles BPQD-RMNVs by wrapping BPQDs with well-biocompatible red blood cell (RBC) membranes (RM). The modification of RBC ensured the “non-enemyization” of nanoparticles, achieving long-lasting circulation *in vivo*. The presence of black phosphorus gives the nanoparticles excellent NIR photothermal properties (conversion efficiency close to 30%). NIR irradiation induces apoptosis in triple-negative breast cancer cells in situ, and the apoptotic tumor cells can recruit DCs and recognize the released neoantigens. This therapeutic strategy, combined with αPD-1 therapy, can further activate CD8^+^T cells to eventually eliminate primary and secondary tumors. In addition to the BPQDs mentioned above, BP sheets of the BP family are also making significant contributions to the biomedical field. It has been found that BP sheets have a very high surface area after stripping and can be used as efficient anti-tumor drug carriers while achieving functions such as PTT, PDT and biomedical imaging. Zhang et al. (144) used the inherent properties of BP to achieve tumor immunotherapy with a BP-based “minimalist” protocol. BP-based PTT kills tumor cells directly, releases tumor-associated antigens, and acts as an immunostimulant to alleviate immunosuppression of the tumor microenvironment. This strategy, combined with αCD47 immune checkpoint blocker, induced polarization of TAMs toward M1, which blocked the interaction of CD47 with SIRPα to improve phagocytosis of macrophages and enhanced the presentation of TAAs. The cases mentioned above related to BP illustrate the influence of the same substance in different self-morphologies on tumor immunotherapy strategies and suggest that biomaterials scientists can modify the nanostructures according to different situations when designing the structures of nanoparticles (153–155).

## Summary

Surgery remains the treatment for most patients with solid tumors at this stage. Unfortunately, attributed to tumor recurrence and metastasis, long-term survival rates for cancer patients are challenging to improve significantly. Tumor immunotherapy, as a newcomer, has become one of the most effective clinical treatment strategies against tumor recurrence and metastasis. However, this “self-interest against self-inflicted disease” option still prevents most patients with solid tumors from benefiting due to the complex physiological and physical barriers at the tumor site. The advent of nanomaterials has challenged the difficulty of poor immunotherapy. Carefully modified nanomaterials can be used alone to enhance immune efficacy or as adjuvants to immunotherapy synergize immunotherapy. Importantly, nanomaterials often have more than a single role in immunotherapy due to their modifiability and their own properties, such as the ability to convert light to heat. In addition to the efficient delivery of long-lasting antitumor drugs to tumor sites, self-assembled nanomaterials can also activate immunity to prevent tumor recurrence by changing their own structure or coupling photosensitizers. In addition to directly ablating and killing tumor cells, the combination of PDT and PTT with immunotherapy can transform cold tumors into hot tumors by modulating the immunosuppressive microenvironment. They can also promote the recognition and killing of immune cells by regulating the oxygen content, metabolism, and release of new antigenic molecules in tumors. Combination therapy through cell membrane camouflage not only escapes clearance by the mononuclear macrophage system, but also allows efficient aggregation of functional components at the tumor site. As an example, nanomaterials modified by immune cell membranes can also domesticate enemy forces to become friendly. Despite the rapid development of nanomaterials in biotherapeutics, we still need to pay attention to the fact that few nanomaterials have received regulatory approval for cancer treatment. The ultimate goal of nanomedicine development is to free human beings from diseases and improve the quality of life of patients. Although its translational application is imminent, it should not be taken lightly. Only by elevating the safety research of nanomedicines to a strategic level can clinical trials be carried out smoothly and patients can benefit from them.

Nanomedicines are exogenous substances of a certain size and their improper use can cause problems such as immune rejection, embolism, liver and kidney failure, factor storm, etc. At present, most of the FDA-approved nanomedicines use liposomes as carriers, and how to make their clinical applications beyond liposomes is also a common problem faced by many disciplines. This is because there are still many limitations and uncertainties, including the clearance of nanomaterials *in vivo* and their short-term and long-term toxicity. Of particular importance is whether their biological effects *in vivo* will impact the body and the consequences. In addition, unlike the single chemical formula of antitumor drugs such as small molecules, we benefit from nanomaterials’ modifiability and multifunctionality. The repeatability of its complex structure and the consistency of its function from batch to batch should also be considered. These are the bottlenecks that limit the translation of nanomaterials to the clinic. In this review, we discuss several nanomaterials for tumor immunotherapy, which function in immunotherapy with their unique advantages and have abysmal implications for effectively addressing the thorny cancer problem. In conclusion, the combination of immunotherapy in the antitumor and nanomaterials field will become closer as the design, synthesis, and application of the two continue to improve. It is worth expecting that immunotherapy based on nanomaterials will shine on it the global problem of the tumor and bring more light to patients.

## Author contributions

SL: Conceptualization, Methodology, Software Data curation, and Funding support. ZC : Writing- Original draft preparation. ZY and RW: Visualization, Investigation, Supervision. KY : Writing- Reviewing and Editing. All authors contributed to the article and approved the submitted version.

## Funding

This work was financially supported by the Fundamental Research Funds for the Central University (LD202110).

## Acknowledgments

This work was supported by the Liaoning Cancer Hospital and Institute (Shenyang) and Harbin Medical University

## Conflict of interest

The authors declare that the research was conducted in the absence of any commercial or financial relationships that could be construed as a potential conflict of interest.

## Publisher’s note

All claims expressed in this article are solely those of the authors and do not necessarily represent those of their affiliated organizations, or those of the publisher, the editors and the reviewers. Any product that may be evaluated in this article, or claim that may be made by its manufacturer, is not guaranteed or endorsed by the publisher.

## Glossary

**Table:**

APCs	antigen-presenting cells
CAR-T	chimeric antigen Q13 receptor-T cell
CCMs	cancer cell membranes
CDT	chemodynamic therapy
CQ	chloroquine
CTLs	cytotoxic T lymphocytes
CR	CPTArginine;
CRA	CR self-assemble
DAMPs	damage-associated molecular patterns
DCs	dendritic cells
Dnase	DNA endonuclease
DOX	doxorubicin
FNAs	functional nucleic acids
GNPs	gold-core
nanoparticles	
GOx	glucose oxidase
GSNO	S-nitrosoglutathione
HA	hyaluronic acid
HAase	hyaluronidase
IAPs	inhibitors of apoptosis proteins
ICB	immune checkpoint blockade
ICD	immunogenic cell death;
LNs	lymph nodes
MB	methylene blue
MDSCs	myeloid-derived suppressor cells
MOF	metal-organic framework
MRI	magnetic resonance imaging;
MSNs	mesoporous silica nanoparticles
MTT	molecular targeted therapy;
NK	natural killer
NPs	nanoparticles
PDA	polydopamine
PDT	photodynamic therapy
PEG	polyethylene glycol
PET	positron emission tomography
PTAs	photothermal conversion agents
PTI	photoimmunotherapy
PTT	photothermal therapy
ROS	reactive oxygen species
RT	radiotherapy
TA	tannic acid
TAAs	tumor-associated antigens;
TAT	transactivator
TIME	tumor immunosuppressive microenvironment;
TLR9	toll-like receptor 9
TME	tumor microenvironment
